# Gellan Gum Fluid Gel System for Controlled‐Delivery of Cytokine‐Licensed MSC‐EVs to Enhance Corneal Repair in Limbal Stem Cell Deficiency

**DOI:** 10.1002/adhm.71402

**Published:** 2026-06-30

**Authors:** Seyedmohammad Moosavizadeh, Ava Dashti, Thomas E. Robinson, Robyn Gaffey, Richard Moakes, Neil Lagali, Liam Grover, Thomas Ritter

**Affiliations:** ^1^ Regenerative Medicine Institute (REMEDI), Discipline of Advanced Therapies School of Medicine University of Galway Galway Ireland; ^2^ Research Ireland Centre For Medical Devices (CURAM) University of Galway Galway Ireland; ^3^ Division of Ophthalmology Department of Biomedical and Clinical Sciences Linköping University Linköping Sweden; ^4^ Healthcare Technologies Institute School of Chemical Engineering University of Birmingham Birmingham UK

**Keywords:** cornea, cytokine licensing, extracellular vesicles, fluid gel, limbal stem cell deficiency, mesenchymal stromal cells, ocular delivery

## Abstract

Corneal diseases such as limbal stem cell deficiency (LSCD) are major causes of blindness, and effective options for severe injury remain limited. Mesenchymal stromal cell extracellular vesicles (MSC‐EVs) have immunomodulatory and regenerative activity, which can be enhanced by cytokine licensing of MSCs with TGF‐β1 and IFN‐γ. A key barrier is achieving sustained, localized ocular delivery. Here, we combine licensed MSC‐EVs with a gellan gum fluid gel (FG), a biocompatible, shear‐thinning viscoelastic system designed to improve residence time versus conventional eyedrops. Licensed MSC‐EVs were isolated by size‐exclusion chromatography and characterized by morphology, size, and marker expression. FG was produced by shear gelation and assessed for transparency, rheology, EV release, and cytocompatibility. EVs were spherical (102 nm) and expressed CD9, CD63, CD81, and TSG101. FG behaved as solid at rest and flowed under shear. The lead formulation released 18% of loaded EVs over 6 h, significantly outperforming comparator gels (*p* < 0.0001), and was non‐toxic to human corneal epithelial cells and keratocytes. Released EVs accelerated closure in an in vitro corneal wound model and reduced epithelial defect and inflammation in a traumatic LSCD model in vivo. Gellan gum FG therefore enables controlled MSC‐EV delivery and shows promise for treating severe corneal disease.

## Graphical abstract

Gellan gum fluid gel (FG) enables controlled ocular delivery of mesenchymal stromal cell (MSC)‐derived extracellular vesicles (EV). Its shear‐thinning, solid‐liquid‐solid rheology ensures prolonged ocular retention. Sustained MSC‐EV release (∼18% in 6 hrs) supports effective EV ocular delivery. FG‐MSC‐EVs accelerate wound healing in limbal stem cell deficiency models. This novel treatment reduces inflammation and preserves native corneal epithelial identity.

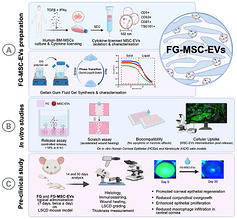

## 1 Introduction

The cornea is the transparent outermost tissue of the eye, vital for both vision and the protection of the deeper ocular structures [1]. The corneal epithelium is part of the ocular immune system and serves as a barrier against pathogens [2]. Beneath it, a thick stromal layer plays a critical role in the cornea's mechanical and optical properties [2]. Corneal regeneration and re‐epithelialization are primarily facilitated by the differentiation and migration of limbal stem cells (LSCs) located in the limbus [3]. Corneal diseases and injuries, such as corneal ulcers and chemical burns, can result in corneal stroma scarring and thinning, as well as partial limbal stem cell deficiency (LSCD), which can ultimately lead to blindness [4, 5].

Corneal and limbal stem cell transplantation (LSCT) are the most common treatments for restoring corneal transparency [6, 7]. However, these procedures often have poor outcomes in severe cases complicated by inflammation of the corneal surface, [8, 9] and require donor tissues, advanced surgical techniques, and pose risks of rejection, along with the potential need for long‐term systemic immunosuppression [8, 10].

Mesenchymal stem/stromal cells (MSCs) and their secreted extracellular vesicles (MSC‐EVs) have demonstrated promising anti‐inflammatory, immunomodulatory, and pro‐regenerative effects for corneal repair and regeneration [11, 12, 13, 14]. MSC‐EVs are nanoscale, lipid‐bilayer particles that carry proteins, lipids, and nucleic acids, facilitating interactions with recipient cells through mechanisms such as endocytosis, membrane fusion, or receptor interactions [15, 16]. These EVs mediate at least in part the therapeutic effects of their parent cells while overcoming challenges associated with traditional cell therapies, including immunogenicity, tumorigenesis, dosage and delivery issues, and unidirectional differentiation [17, 18]. Preconditioning or “licensing” MSCs with bioactive compounds, or by adjusting their growth conditions, or physical stimuli can enhance their therapeutic potential [19, 20]. Alternatively, treating MSCs with pro‐ and/or anti‐inflammatory cytokines such as interferon‐gamma (IFN‐γ) [21] and transforming growth factor‐beta 1 (TGF‐β1) [22], either individually or in combinations (TGF‐β1 + IFN‐γ) [14, 23], significantly boosts the immunomodulatory and anti‐inflammatory effects of the MSCs and their secreted EVs [23, 24]. The anti‐inflammatory activity occurs through inhibiting pro‐inflammatory THP‐1 macrophage activation, as the standard assay [25], and promoting an anti‐inflammatory phenotype, with reduced secretion of tumour necrosis factor‐alpha (TNF‐α) and interleukin‐1 beta (IL‐1β), increased IL‐10 production, and decreased nitric oxide (NO) levels [24]. These immunomodulatory activities subsequently enhance the efficacy of MSCs and their secreted EVs within in vitro and in vivo injury models [24, 26, 27]. Despite their potential, however, several challenges hinder the clinical application of MSC‐EVs in the eye, including delivery methods, in vivo biodistribution and off‐target localization [28, 29, 30]. Eye drop systems, while widely used, suffer from rapid clearance [31, 32].

As an alternative strategy, biomaterial‐based drug delivery using fluid gels represents a promising approach [33, 34]. Unlike traditional hydrogels with continuous networks, fluid gels are composed of microgel particles created by applying shear during the gelation process [35]. This microstructure can be controlled by adjusting gelation kinetics and shear conditions, allowing customization of mechanical properties without altering the chemical composition, an important advantage for clinical application [33, 36]. Weak interactions between particles lead to viscoelastic, solid‐like behavior while at rest. However, under shear forces, such as during blinking or application, the network breaks down and flows, only to re‐establish itself once the shear force is removed [33, 37]. This solid‐liquid‐solid transition enables the fluid gel to adapt to the ocular surface while prolonging therapeutic agent residence time through gradual release [37, 38].

Gellan gum, a microbial polysaccharide from Sphingomonas elodea, is prominent in pharmaceutical applications due to its biocompatibility, mucoadhesive properties, and tuneable gelation behavior [39, 40]. Its linear tetra saccharide structure forms hydrogels through ionic crosslinking, exhibiting favourable mechanical and rheological properties for controlled drug delivery [33, 41]. In ophthalmology, gellan gum prolongs therapeutic residence time by binding water and adhering to corneal mucosa, enhancing drug efficacy [33, 39]. Gellan gum's transparency and non‐cytotoxicity further support its use ophthalmic drug delivery applications particularly as a fluid gel system [42, 43].

Collectively, these properties position gellan gum and its fluid gel materials as versatile and effective platforms for advanced ocular delivery systems for MSC‐EVs. Accordingly, the aim of this study was to develop a gellan gum‐based fluid gel system for cytokine‐licensed MSC‐EV delivery to the corneal surface in a controlled manner, to enhance wound healing and immunomodulatory properties by increasing ocular surface retention time while providing mechanical support and unique rheological features.

## 2 Materials and Methods

### 2.1 Cytokine‐Licensed MSC‐EVs Isolation and Characterization

The extracellular vesicles (EVs) were isolated from human bone marrow mesenchymal stromal cells (MSCs) concentrated xeno‐free [44] cytokine licensing growth medium (Method S1). To isolate cytokine‐licensed MSC‐derived extracellular vesicles (MSC‐EVs), the concentrated medium underwent size exclusion chromatography (SEC) with q‐EV10 Gen 2 columns (iZON) as per the manufacturer's instructions. Fractions containing MSC‐EVs were pooled, aliquoted in 0.1 µm filtered PBS, and stored at ‐80°C for further analysis.

MSC‐EVs were characterized according to MISEV‐2023 guidelines [45]. Size distribution and concentration were assessed by diluting the 100 nm QC beads (Malvern Zetasizer, Worcestershire, UK) and the isolated MSC‐EVs in ultra‐pure 0.1 µm filtered water (1:1000) and analysing them via nanoparticle tracking analysis (NTA) with the Nanosight NS500 (Malvern Zetasizer, Worcestershire, UK). The morphology of MSC‐EVs was examined by adsorbing them onto Formvar/carbon‐coated grids, staining with 2% phosphotungstic acid (PTA) (Sigma‐Aldrich, cat#: HT152‐250ML), air‐drying the grids, and imaging using a Hitachi 7500 transmission electron microscope (TEM) at 100.0 kV accelerating voltage and 50,000× direct magnification. Surface biomarkers were analysed through high resolution flow cytometry (Northern Lights 3000, Cytek). MSC‐EVs were stained with antibodies for CD9‐APC (BioTechne R&D Systems, cat#: NB500‐327APC), CD63‐PE (BD Biosciences, cat#: 557305), and CD81‐FITC (BD Pharmingen, BD Biosciences, cat#: 551108) at a 1:20 concentration in 0.1 µm filtered PBS. Apogee size calibration beads (Apogee Flow Systems, UK), 0.1 µm filtered PBS, and unstained MSC‐EVs were analysed as the control groups, which were used to set forward scatter (FSC) and side scatter (SSC) voltages, gate background noise, and confirm antibody staining of the MSC‐EVs. For immunoblotting, samples were prepared on ice by mixing 25 µL of whole‐cell lysate or MSC‐EV solution with 5 µL of Laemmli buffer supplemented with β‐mercaptoethanol (9:1). Samples were vortexed, centrifuged, denatured at 95°C for 5 min, and immediately cooled on ice. Proteins were resolved on 12% precast polyacrylamide gels (Bio‐Rad) using Tris–Glycine running buffer (Bio‐Rad). A molecular weight standard (5 µL) and prepared samples (25 µL) were loaded, and electrophoresis was performed at 180 V for 30 min. Proteins were subsequently transferred to polyvinylidene difluoride (PVDF) membranes using a semi‐dry transfer system (Trans‐Blot Turbo, Bio‐Rad) at 25 V for 18 min. Membranes were blocked for 1 h at room temperature in TBS‐T (TBS with 0.1% Tween‐20) containing 10% non‐fat dry milk (NFDM), followed by two washes in TBS‐T (10 min each, 4°C). Primary antibody incubation was carried out overnight at 4°C with gentle rocking using rat anti‐TSG101 (1:1000; BioLegend, Cat. No. 934301) and rat anti‐Calnexin (1:1000; BioLegend, Cat. No. 699401). After three washes in TBS‐T (10 min each, 4°C), membranes were incubated for 1 h at room temperature with HRP‐conjugated goat anti‐rat IgG (1:5000; BioLegend, Cat. No. 405405) diluted in TBS‐T containing 3% NFDM. Blots were washed three times in TBS‐T (10 min each) and developed using enhanced chemiluminescence (ECL; Clarity, Bio‐Rad) for 5 min. Protein bands were visualized using a Vilber imaging system (Vilber, France).

### 2.2 Gellan Gum Fluid Gel Synthesis

Gellan gum fluid gel formulations (Table 1) were prepared by dissolving sodium chloride (NaCl) (Sigma‐Aldrich, UK) in varying amounts to achieve final NaCl concentrations of 1, 5, 10, or 20 mM in ultra‐pure 0.1 µm filtered water at 90°C under continuous stirring (1000 rpm) in a cell stirrer flask to ensure complete dissolution. Once fully dissolved, low acyl gellan gum (Kelco gel CG, Azelis, UK) powder was added in specific amounts to create solutions with final polymer concentrations of 0.5%, 0.75%, 1%, and 1.5% (w/v) under continuous stirring at 90°C to ensure complete dissolution. Then, the mixture was gradually cooled while being stirred at 1000 rpm using a magnetic stirrer to maintain uniformity to a final temperature of 20°C during which the gelation process was observed/occurred. After preparation, the fluid gel samples were removed, stored in sterile conditions, and kept at 4°C for future use.

**TABLE 1:** Experimental design with final concentrations of gellan gum polymer and NaCl as the cross‐linker.

Formulation	A	B	C	D	E	F	G	H	I	J	K	L	M	N	O	P
Gellan gum con. [%w/v]	0.5	0.5	0.5	0.5	0.75	0.75	0.75	0.75	1	1	1	1	1.5	1.5	1.5	1.5
NaCl con. [mM]	1	5	10	20	1	5	10	20	1	5	10	20	1	5	10	20

To load the gellan gum fluid gels with MSC‐EVs, the fluid gels were warmed up using water bath at 37°C, then the MSC‐EVs were mechanically mixed with the gellan gum fluid gel using a magnetic stirrer (500 rpm) to ensure even distribution. The resulting MSC‐EVs loaded gellan gum fluid gel formulation (FG‐MSC‐EVs) was then ready for later applications or analysis.

### 2.3 Gellan Gum Fluid Gel Characterization

Optical transparency measurements were conducted using Spark Cyto UV/visible spectrophotometer (Tecan, Switzerland). Ultra‐pure 0.1 µm filtered water and a 1% (w/v) opaque agarose gel were used as positive and negative controls, respectively. Gellan gum fluid gel samples were placed in 1 mm cuvettes, and the absorbance at a wavelength of 600 nm was recorded. Each sample was tested in triplicate, and the results were averaged. Standard error bars, representing the standard deviation, were included in the data presentation.

The rheological properties of the gellan gum fluid gel formulations were evaluated for both linear and non‐linear behavior using a Kinexus Ultra+ rheometer (Malvern Panalytical, UK). The instrument was fitted with a 40 mm polished parallel plate geometry, operating at 20°C with a 1 mm gap height. Prior to testing, the samples were loaded onto the rheometer and allowed to equilibrate for 5 min. Amplitude sweeps were conducted in stress control mode, using stress values ranging from 0.001 to 100 Pa at a fixed frequency of 1 Hz. Frequency‐dependent measurements were carried out at a constant stress level within the linear viscoelastic region (LVeR) for all samples (0.05 Pa), covering a frequency range from 0.01 to 10 Hz. Dynamic viscosity was measured in stress‐controlled mode between 0.01 and 100 Pa over a 10‐min ramp.

### 2.4 Gellan Gum Fluid Gel In Vitro Biocompatibility

The in vitro biocompatibility of selected gellan gum fluid gel formulations, FG1 (0.75% gellan gum with 10 mM NaCl), FG2 (1% gellan gum with 5 mM NaCl), and FG3 (1% gellan gum with 10 mM NaCl), was assessed by analysing their effects on human corneal keratocytes (HCK) (Cell Applications Inc., cat#: 632‐05a) and immortalized human corneal epithelial cells (HCEpi) [46, 47] using a multiplex fluorescent assay. HCK and HCEpi cells were seeded at 10,000 and 25,000 cells/cm^2^ in 48‐well plates, respectively. The HCEpi cells were cultured in Dulbecco's Modified Eagle Medium (DMEM) + GlutaMAX‐1 (1 g/L glucose) (Gibco, ThermoFisher Scientific, cat#: 21885‐025) with 5% fetal bovine serum (GE Healthcare, FisherScience, Ref: 10309433), 1% penicillin/streptomycin (Gibco, ThermoFisher Scientific, cat#: 15140122), 40 ng/mL insulin (Roche, cat#: 11376497001), and 0.52 µg/mL hydrocortisone (Sigma‐Aldrich, cat#: H0888). HCK cells were cultured in DMEM + GlutaMAX‐1 (4.5 g/L glucose) (Gibco, ThermoFisher Scientific, cat#: 31966‐021) supplemented with 10% fetal bovine serum, 1% penicillin‐streptomycin, 1x non‐essential amino acids (Gibco, ThermoFisher Scientific, cat#: 11140‐035), and 20 mM L‐glutamine (Gibco, ThermoFisher Scientific, cat#: 25030–081). Once confluent, the media was removed, and cells were rinsed with PBS. Subsequently, 300 µL of media was added to each group: (1) media only, (2‐4) 50 µL of the selected gellan gum fluid gel formulations, and (5) 1 µM staurosporine (Sigma‐Aldrich, cat#: 62996‐74‐1)‐supplemented media solution. The media only group was used as the negative control and the staurosporine‐supplemented media group was used as the positive control as it is known to induce apoptosis in cells [48]. The cells were incubated at 37°C with 5% CO_2_ for 6 h, washed twice with PBS, and fluorescently stained on ice with a mixture of 1 µM Hoechst 33342 (ThermoFisher Scientific, cat#: 62249) for all cells, 0.1 µM YO‐PRO‐1 (Invitrogen, ThermoFisher Scientific, cat#: Y3603) for apoptotic cells, and 1 µg/mL propidium iodide (PI) (Invitrogen, ThermoFisher Scientific, cat#: P1304MP) for necrotic cells for 15 min in controlled light conditions. After washing twice with PBS, fluorescent images were captured using the Spark Cyto Cell Imager (Tecan, Switzerland) at 461 nm, 509 nm, and 618 nm emission wavelengths for Hoechst, YO‐PRO‐1, and PI, respectively.

### 2.5 MSC‐EVs Release Profile From FG Systems

The in vitro release profile of MSC‐EVs from the gellan gum fluid gel formulations, FG1, FG2, and FG3 was assessed using dispersion method. MSC‐EVs were fluorescently labelled with CellTrace Far Red (CTFR; Invitrogen, ThermoFisher Scientific, cat#: C34564) in 0.1 µm filtered PBS at a final concentration of 35 nM, prepared in 1.5 mL screw‐top Eppendorf tubes. The samples were incubated at 37°C for 2 hr and subsequently purified using qEV Original 35 nm Gen 2 SEC columns (iZON) to eliminate any unbound CTFR. The fractions containing CTFR‐labelled MSC‐EVs were pooled, analysed by NTA, and stored at 4°C. The standard equation and R‐square for CTFR‐labelled MSC‐EV concentrations were measured by evaluating the fluorescence intensity of a serial dilution of 1 × 10^9^, 5 × 10^8^, 1 × 10^8^, 5 × 10^7^, 1 × 10^7^, 5 × 10^6^, 1 × 10^6^, and 0.0 CTFR‐labelled MSC‐EVs/mL.

Subsequently, 1 × 10^9^ CTFR‐labelled MSC‐EVs were loaded into 500 µL of the selected gellan gum fluid gel formulations. The resulting gellan gum fluid gels containing CTFR‐labelled MSC‐EVs were then placed in a 24‐well plate and allowed to rest for 1 h at room temperature under controlled light conditions. Following this, 1 mL of 0.1 µm filtered PBS was gently added to each well as a release sink, and the plates were incubated at 32°C for 6 h on a shaker incubator set to 15 linear shakes per minute [49], simulating ocular surface and blinking conditions. At 1, 2, 4, and 6‐h post‐release time points, 200 µL samples were collected from each well and immediately replaced with an equal volume of 32°C 0.1 µm filtered PBS. The samples were subsequently analysed using Spark Cyto Cell Imager (Tecan, Switzerland) to quantify the cumulative release of CTFR‐labelled MSC‐EVs from the selected gellan gum fluid gel formulations, with measurements taken at an emission wavelength of 650 nm, and concentrations calculated based on the standard calibration.

### 2.6 MSC‐EVs Uptake by HCEpi and HCK Cells

The in vitro cellular uptake of MSC‐EVs released from gellan gum fluid gel was assessed using confocal microscopy. The lead FG formulation, FG2 (1% gellan gum with 5% NaCl), was loaded with 1 × 10^9^ fluorescently labelled MSC‐EVs per milliliter. Human corneal epithelial (HCEpi) and keratocyte (HCK) cells were treated with the FG loaded with fluorescently labelled MSC‐EVs and incubated at 37°C with 5% CO_2_ for 6 h. After incubation, the FG was removed, and the cells were cultured for an additional 18 h, followed by two washes with PBS. Cells were then fixed with 500 µL of 1% methanol‐free formalin (Sigma) for 15 min at room temperature and washed twice with PBS. Permeabilization was performed using 500 µL of 0.1% Triton X‐100 (Sigma) for 15 min at room temperature, followed by two more PBS washes. F‐actin filaments were stained with Flash Phalloidin Green 488 (Abcam) for 30 min at room temperature in the dark, and the cells were again washed twice with PBS. To visualize cell nuclei, one drop of ProLong Glass Antifade Mountant with NucBlue Stain (Invitrogen) was added to each slide, which was then covered with a coverslip and stored in a light‐protected environment for 1 h to allow the mountant to harden. Finally, the cells were imaged using a confocal microscope (FLUOVIEW FV3000, Olympus) to evaluate the uptake of fluorescently labelled MSC‐EVs following their release from the gellan gum fluid gel.

### 2.7 FG‐MSC‐EVs Scratch Wound Healing In Vitro

HCK and HCEpi were employed to investigate the wound healing properties of the MSC‐EVs after releasing from the gellan gum fluid gel. The cells were cultured in 12 well‐plates. Once the cells reached 95% confluency, the medium was removed, and a linear scratch was made across the centre of each well using a sterile 200 µL pipette tip (Cruinn Diagnostics, Ireland). The wells were then rinsed twice with PBS to eliminate any cell debris. Following this, 1 mL of either standard medium (control), 1 × 10^9^ MSC‐EVs, or FG‐MSC‐EVs with 1 × 10^9^ MSC‐EVs was added to each well. Treatments were administered only once immediately after the scratch was introduced. The cells were incubated at 37°C in a humidified atmosphere with 5% CO_2_. Bright field images were captured at 0, 3, 6, and 20 h and 0, 18, and 24 h post‐scratch for the HCEpi and HCK cells, respectively, using an Olympus CKX53 inverted fluorescent microscope. The borders of the scratch were manually drawn using the polygon selection tool in ImageJ v1.54G [50]. To measure scratch width and area, the Wound Healing Size Tool (Updated) plugin for ImageJ was used [50, 51].

### 2.8 Pre‐Clinical Limbal Stem Cell Deficiency (LSCD) Model

#### 2.8.1 Animal Model

In this study, 5‐week‐old homozygous wild‐type mice with a 129S1/SvImJ genetic background were bred and housed under standardized conditions at Linköping University, in accordance with previously described protocols [52]. To investigate the therapeutic effect of MSC‐EVs released from the FG, 12 mice were assigned to the FG‐only group and 12 to the FG loaded with MSC‐EVs group (FG‐MSC‐EVs). All experiments complied with ethical guidelines from Linköping University's regional ethics committee (Permit No. 06826‐2024) and the Association for Research in Vision and Ophthalmology (ARVO) standards for animal use in vision research.

#### 2.8.2 Corneal Epithelial Wounding Approach

All mice were anesthetized by intraperitoneal injection with a combination of ketamine (100 mg/kg; stock concentration 50 mg/mL; Orion Pharma AB) and xylazine (11.2 mg/kg; stock concentration 20 mg/mL; Pfizer AB). Prior to corneal injury, topical anaesthesia was administered using 0.5% tetracaine‐HCl eye drops (Bausch & Lomb UK Ltd.). Additionally, the right eyes received a mixture of 4% w/v lidocaine hydrochloride and 0.25% w/v fluorescein sodium eye drops (Bausch & Lomb UK Ltd.) for 1 min, after which excess solution was removed by two washes with phosphate‐buffered saline (PBS; Gibco, #20012‐027). The corneas were subsequently imaged using a slit‐lamp camera. Corneal epithelial wounds were created using a sterile 0.5‐mm Algerbrush II rust ring removal tool (Alger Inc., TX, USA, cat #BR2‐5). The epithelial layer was carefully removed from the entire cornea within a defined circular area (3 mm diameter), avoiding penetration into the stromal layer. Following initial epithelial removal, 30% ethanol was applied to the wounded area for 30 s to facilitate epithelial cell detachment. A second gentle pass with the Algerbrush was performed to ensure complete epithelial removal. After the injury, the corneas were thoroughly rinsed with sterile water. Fluorescein solution was reapplied to the corneal surface, and a slit‐lamp camera was used to verify complete epithelial removal. Slit‐lamp images of the untreated model at different time points, with and without fluorescein staining, are shown in Figures S1A and S1B. To minimize infection risk, antibiotic eye ointment (Fucithalmic 10 mg/g; Amdipharm UK Ltd.) was applied daily to the injured eyes for three consecutive days. For anaesthesia reversal, mice received subcutaneous injections (200 µL) of atipamezole (1.8 mg/kg), prepared by diluting Antisedan (5 mg/mL; Orion Pharma AB) 1:22.2 in sterile PBS to achieve a final concentration of 0.225 mg/mL. Buprenorphine (0.12 mg/kg) was co‐administered for analgesia, prepared by diluting Temgesic (0.3 mg/mL; Indivior) 1:20 in the same PBS‐atipamezole solution, resulting in a final buprenorphine concentration of 0.015 mg/mL. Following treatment, mice recovered in warmed cages until fully awake. Analgesic treatment continued with subcutaneous pain relief injections administered every 12 h for a total duration of three days.

#### 2.8.3 FG‐MSC‐EVs Administration

Following corneal epithelial wounding in the right eyes, mice received topical administration of (i) FG or (ii) FG‐MSC‐EVs with 1 × 10^9^ MSC‐EVs (10 µL per drop) twice daily for one week. After completing this one‐week treatment period, topical application was discontinued. The mice were then maintained without further treatment for three weeks to evaluate efficacy of treatment, after which the mice were euthanized, and ocular tissues were harvested for later analysis (Figure 5A).

#### 2.8.4 In Vivo Ocular Imaging

Corneal examinations were conducted using a rodent‐specific slit‐lamp biomicroscope (Micron III, Phoenix Technologies, USA) and fluorescein staining. A solution containing 4% w/v lidocaine hydrochloride and 0.25% w/v fluorescein sodium (Bausch & Lomb UK Ltd.) was applied to the eye for 1 min, after which excess dye was removed with two washes of phosphate‐buffered saline (PBS; Gibco, #20012‐027). Slit‐lamp photography was employed with white light illumination to visualize and quantify the extent of corneal vascularization, and with a blue light filter to visualize the wound area. In vivo ocular imaging was conducted at baseline (0 h), 2 weeks, and 4 weeks post‐injury to evaluate disease progression and corneal healing LSCD in mouse corneas was graded based on the extent of epithelial involvement (corneal clarity and/or vascularization). Corneas were classified as Stage I when the central 1 mm zone retained normal epithelium, Stage II when the central 1 mm zone was affected, and Stage III when the entire corneal surface was involved [53]. Additionally, central corneal thickness was measured using the iVue 100 optical coherence tomography (OCT) system (Optovue Inc., USA) set to corneal pachymetry mode. Quantitative analyses of the OCT images were performed using integrated OCT analysis software (version 3.2.1.8).

#### 2.8.5 Corneal Sample Processing

At the conclusion of the 4‐week experimental period, whole eyes were extracted and processed for histological analysis. Following extraction, eyes were thoroughly rinsed with PBS, fixed in a 4% paraformaldehyde (PFA) (Thermo Scientific, #10424131) solution at 4°C for 2 h, and subsequently stored in 70% ethanol pending further processing and embedding into paraffin blocks. Immunohistochemical analyses were performed on 5 µm‐thick paraffin‐embedded tissue sections. Comprehensive details regarding corneal sampling and preparation protocols have been previously described [52].

#### 2.8.6 Tissue Staining, Confocal Fluorescence Microscopy, and Quantitative Image Analysis

Tissue sections underwent standard histological processing, including antigen retrieval using an automated DAKO PT Link 200 system, permeabilization with 0.1% Triton X‐100 in PBS, and blocking with PBS containing 3% BSA, 10% goat serum, and 0.1% Tween‐20 for 1 h at room temperature. Primary antibodies against MUC5AC (Invitrogen, #MA5‐12178; 1:50), Ki‐67 (Abcam, #ab16667; 1:100), F4/80 (Bio‐Rad, #MCA497R; 1:100), IL‐1β (Invitrogen, #P420B), and KRT12 (Abcam, #ab185627; 1:100) were incubated overnight at 4°C. Sections were then incubated with secondary antibodies—goat anti‐rabbit Alexa Fluor 647 (Invitrogen, #A32733; 1:400) and goat anti‐mouse Alexa Fluor 647 (Abcam, #ab150115; 1:400)—for 2 h at room temperature, followed by nuclear counterstaining with Hoechst 33342 (Invitrogen, #25811646; 1:1000). Slides were mounted using ProLong Gold Antifade reagent (Invitrogen, #P36930).

Imaging was performed using a Zeiss LSM 800 confocal microscope equipped with a 20× objective under identical exposure and acquisition settings for all samples. For histological assessment, standard H&E staining was conducted, and brightfield images were captured using the same microscope at 20× magnification.

Morphometric analyses were performed using ImageJ software (version 1.54, FIJI distribution). For epithelial cell density quantification, the Cell Counter function was used to manually count epithelial cells in representative H&E‐stained images, and the counts were normalized to the corresponding corneal length. Quantitative fluorescence analysis was conducted on 8‐bit grayscale TIFF images exported from each fluorescence channel. Consistency in fluorescence intensity measurements was ensured by acquiring all images under identical gain settings and performing all immunostaining procedures concurrently. Regions of interest (ROIs) of uniform size (100 × 100 µm) were selected within representative cell‐containing areas, and the mean gray value of each ROI was measured to determine fluorescence intensity.

### 2.9 Statistical Analysis

All statistical analyses were performed using GraphPad Prism software 8.0 (GraphPad Software, Inc., San Diego, CA, USA). Statistical significance was determined at *p* < 0.05. One way ANOVA analysis was employed for the transparency analysis followed by Šídák's, in vitro biocompatibility analysis followed by Dunnett's, MSC‐EVs release assay analysis followed by Tukey's, and in vitro scratch assay analysis followed by Tukey's multiple comparisons test. For the in vivo study data analysis, one‐way ANOVA was used to compare vascularized and wounded areas, as well as corneal thickness, across time and groups. Two‐way ANOVA was employed for the analysis of LSCD grading, epithelial cell density, and fluorescence intensity measurements across time and groups. A p‐value < 0.05 was considered statistically significant. Results are presented as mean ± standard deviation (SD).

## 3 Results

### 3.1 Cytokine‐Licensed MSC‐EVs Isolation and Characterization

**FIGURE 1:**
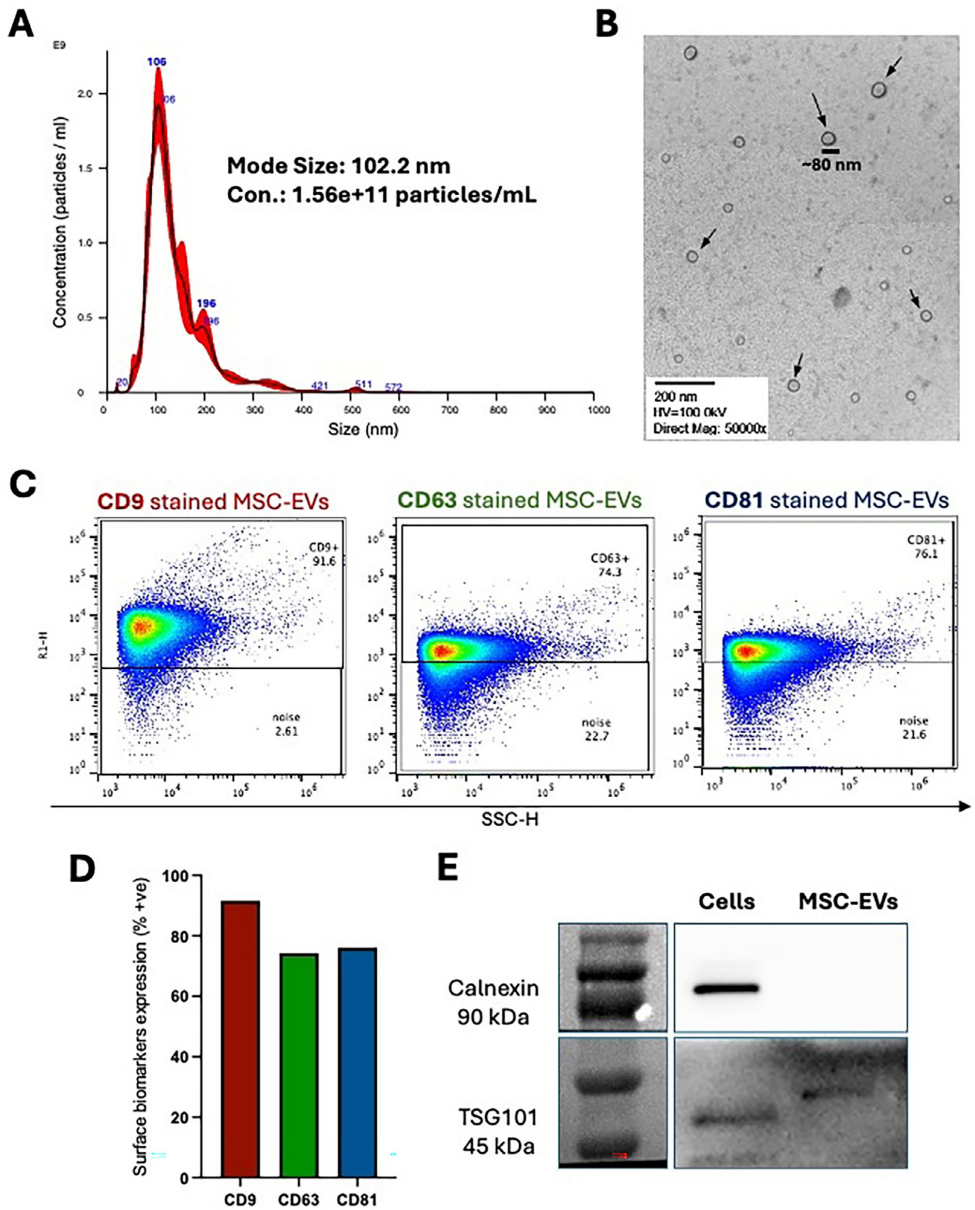
Comprehensive characterization of cytokine‐licensed MSC‐EVs by NTA, TEM, flow cytometry, and western blot analysis. (A) NTA analysis of the size distribution, mode size, and concentration of MSC‐EVs, (B) TEM images of MSC‐EVs with 50,000x direct magnification indicated by black arrows, (C) Flow cytometry analysis of CD9, CD63, and CD81 stained MSC‐EVs, (D) percentage of positive MSC‐EVs for CD9, CD63, and CD81 surface biomarkers expression, and (E) western blot analysis showing the expression of TSG101 (45 kDa) as a cytosolic marker and Calnexin (90 kDa) as a negative marker for MSC‐EVs. All results represent three independent experiments.

Cytokine‐licensed MSC‐EVs were isolated by size exclusion chromatography (SEC) technique using iZON q‐EV10 Gen 2 columns, from human bone marrow MSC concentrated conditioned medium. The isolated MSC‐EVs, along with 100 nm QC beads (Figure S2A), were characterized for their size distribution and concentration through nanoparticle tracking analysis (NTA). The analysis revealed that the MSC‐EVs exhibited a mode size of 101.2 nm and a concentration of 1.56 × 10^1^ ^1^ particles/mL (Figure 1A). The morphology of MSC‐EVs was examined using transmission electron microscopy (TEM) at a magnification of 50,000x, revealing a spherical structure within the desired size range of approximately 100 nm (Figure 1B). Surface biomarker expression on MSC‐EVs was assessed with NL3000 high‐resolution flow cytometer. The side scatter (SSC) and forward scatter (FSC) voltages were calibrated using Apogee size calibration beads. Background noise was gated by running 0.1 µm filtered PBS, and unstained MSC‐EVs served as a negative control, showing that 99.5% of the events fell within the noise gate (Figure S2B). Subsequently, MSC‐EVs were stained with specific antibodies and analysed using the acquisition on the NL3000 flow cytometer (Figure 1C). The results indicated that 91.6%, 74.3%, and 76.1% of the MSC‐EVs were positive for CD9, CD63, and CD81, respectively (Figure 1D). Western blotting was used to assess MSC‐EVs for the presence of TSG101, a cytosolic marker, and Calnexin, a negative control marker. TSG101 (45 kDa) was detected in MSC‐EVs samples. In contrast, Calnexin (90 kDa) was not detected in MSC‐EVs, whereas it was clearly present in whole‐cell lysates (Figure 1E).

### 3.2 Gellan Gum Fluid Gels Characterization and Selection

**FIGURE 2:**
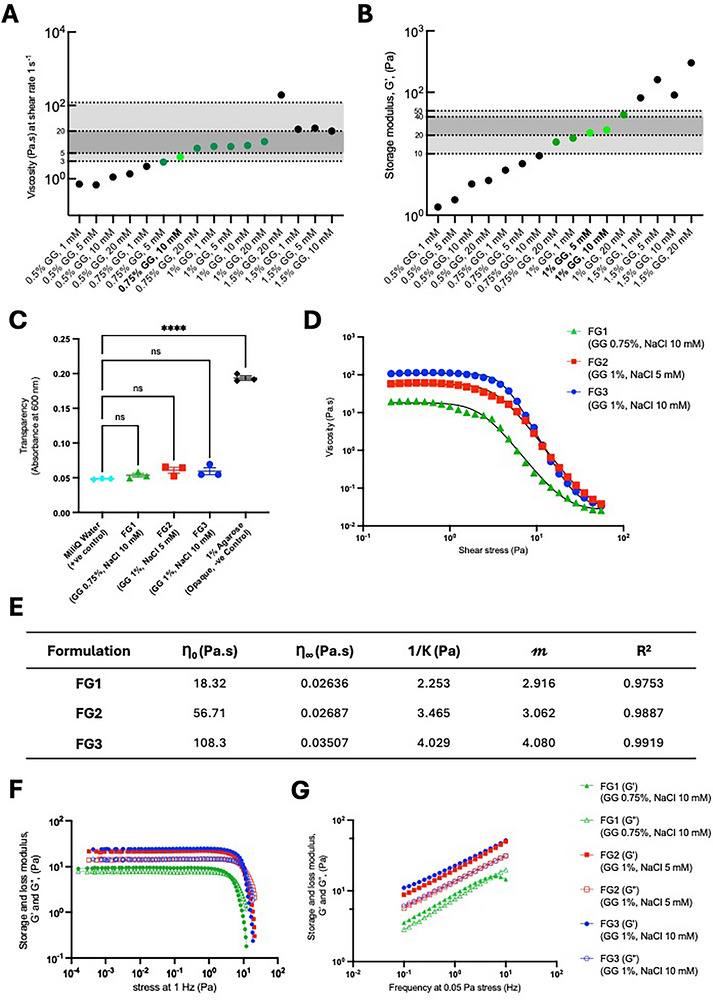
Selection and Physicochemical characterization of gellan gum fluid gel formulations (FG1, FG2, and FG3) by rheological, optical, and statistical analyses. Selection of the gellan gum fluid gel formulations with respect to the ranges (grey lines) obtained from commercially available products, (A) based on the viscosity (Pa.s) range at 1 s‐1 shear stress, (B) based on the storage modulus, G’ (Pa) range at rest. (C) transparency analysis of the selected gellan gum fluid gel formulations, FG1, FG2, and FG3 by their absorbance at 600 nm using UV Spectroscopy, (D) FG1, FG2, and FG3 viscosity profile, (E) Table of data obtained from fitting the flow profiles of the FG1, FG2, and FG3 to the Cross model, where η0 (Pa.s) is the zero‐shear viscosity, η∞ (Pa.s) is the infinite shear viscosity, 1/K (Pa) is the critical shear value to induce thinning, 𝓶 is the thinning index, and R2 is the statistical measure of fitting, (F) Amplitude sweep, stress controlled, at 1 Hz frequency for FG1, FG2, and FG3, and (G) Frequency sweep analysis of FG1, FG2, and FG3 at 0.05 Pa stress. All data presented is the average on n = 3, with error bars demonstrating the 95% confidence interval. Statistical analysis was conducted using one‐way ANOVA with Šídák's multiple comparisons test, where significance denoted as **** *p* < 0.0001.

Gellan gum fluid gel formulations were synthesized using the shear gelation technique, as previously described [35, 36, 54]. In this process, shear forces were applied to a polymer solution undergoing a sol‐gel transition, leading to the formation of a particulate suspension rather than a uniform, continuous network. Each formulation was characterized for transparency, viscosity, frequency sweep, and amplitude sweep (Figure S3). Selected gellan gum fluid gel formulations were identified by employing the ranges previously reported by Grover et al. [55], established from characterization of seven different commercially available eyedrops/ointments for their storage modulus (G’) at rest and viscosity at a given shear rate thinning behavior, complemented by ophthalmologists opinions shown by grey lines in Figure 2A,B. The selected formulations resulting from evaluating the viscosity behavior of the gellan gum fluid gels at 1 s^−1^ shear rate compared to the commercial products, was found to be 0.75% gellan gum fluid gel crosslinked with 10 mM NaCl (FG1) (Figure 2A). Based on the measured storage modulus (G’) of the commercial eye drops at rest, two additional formulations of gellan gum fluid gels were selected: 1% gellan gum crosslinked with 5 mM NaCl (FG2) and 1% gellan gum crosslinked with 10 mM NaCl (FG3) (Figure 2B).

Transparency of the gellan gum fluid gel formulations was assessed by measuring the absorbance at a wavelength of 600 nm using UV spectroscopy. The results indicated no significant difference in absorbance and transparency between the selected gellan gum fluid gel formulations compared to the positive control, ultra‐pure 0.1 µm filtered water (Figure 2C). The selected gellan gum fluid gel formulations viscoelasticity was studied for both linear and non‐linear rheology (Figure 2D‐G). Non‐linear rheology reveals the fluid gel's ability to flow at large deformations like injection. Figure 2D showed all selected fluid gel systems shear thinning profiles closely fitted to the modified‐Cross model using GraphPad Prism (version 10.1.0) [56], Equation (1), which provides a means of comparing the systems (Table in Figure 2E).

(1) $$\eta =\eta \infty +\frac{\eta 0-\eta \infty }{1+{\left(K\sigma \right)}^{m}}$$

where η is the shear viscosity (Pa.s), σ is the shear stress (Pa), η_0_ (Pa.s) is the zero‐shear viscosity, η∞ (Pa.s) is the infinite shear viscosity, 1/K (Pa) is the critical shear stress value to induce thinning, and 𝓶 (‐) is the thinning index.

The critical shear stress required to induce flow (1/K) for FG1 was 2.253 (Pa) increased to 3.465 (Pa), and 4.029 (Pa) for the FG2 and FG3, respectively, showing the effect of gellan gum polymer concentration in the fluid gels behavior. FG3 revealed the highest thinning index (𝓶), while the values for FG1 and FG2 were similar, which closely correlate with the amplitude data shown in Figure 2F. The trend in zero shear viscosity (η_0_) for the fluid gels correlates with other rheology data showing that FG3 has the highest viscosity with 108.3 (Pa.s) zero shear viscosity, decreasing to 56.71 (Pa.s) for FG2, and 18.32 (Pa.s) for FG1. η0 (Pa.s) suggests that different FG formulations represent different level of structuring at zero‐shear viscosity, but at high shear, infinite shear viscosity, once these structures are broken, FG1, FG2, and FG3, they all act similarly (η∞ (Pa.s)) (Figure 2E). Linear rheology, or small deformation analysis, was conducted to assess the behavior of fluid gels at rest. The selected fluid gels were characterized for their storage modulus (G′) and loss modulus (G′′) at a frequency of 1 Hz (Figure 2F). The results indicated that the selected fluid gels exhibited a similar trend, with G′ dominating G′′ within the linear viscoelastic region (LVeR). As the applied stress increased, the systems moved beyond the linear viscoelastic region (LVeR) leading to a transition toward a loss‐dominated response (G″ > G′). Notably, this crossover occurred at higher stress levels (Pa) for FG2 and FG3 compared to FG1 (Figure 2F). Frequency‐dependent measurements were conducted at a constant stress level within the LVeR, revealing that the fluid gels exhibited similar behavior at lower frequencies, with G′ predominating over G′′. However, at higher frequencies, FG3 behaved like a viscoelastic liquid, characterized by loss modulus dominance (Figure 2G). In contrast, FG1 and FG2 maintained a gel‐like behavior throughout the frequency range (0.01 to 10 Hz), with the storage modulus consistently surpassing the loss modulus (Figure 2G).

### 3.3 Gellan Gum Fluid Gel Demonstrates In Vitro Biocompatibility

**FIGURE 3:**
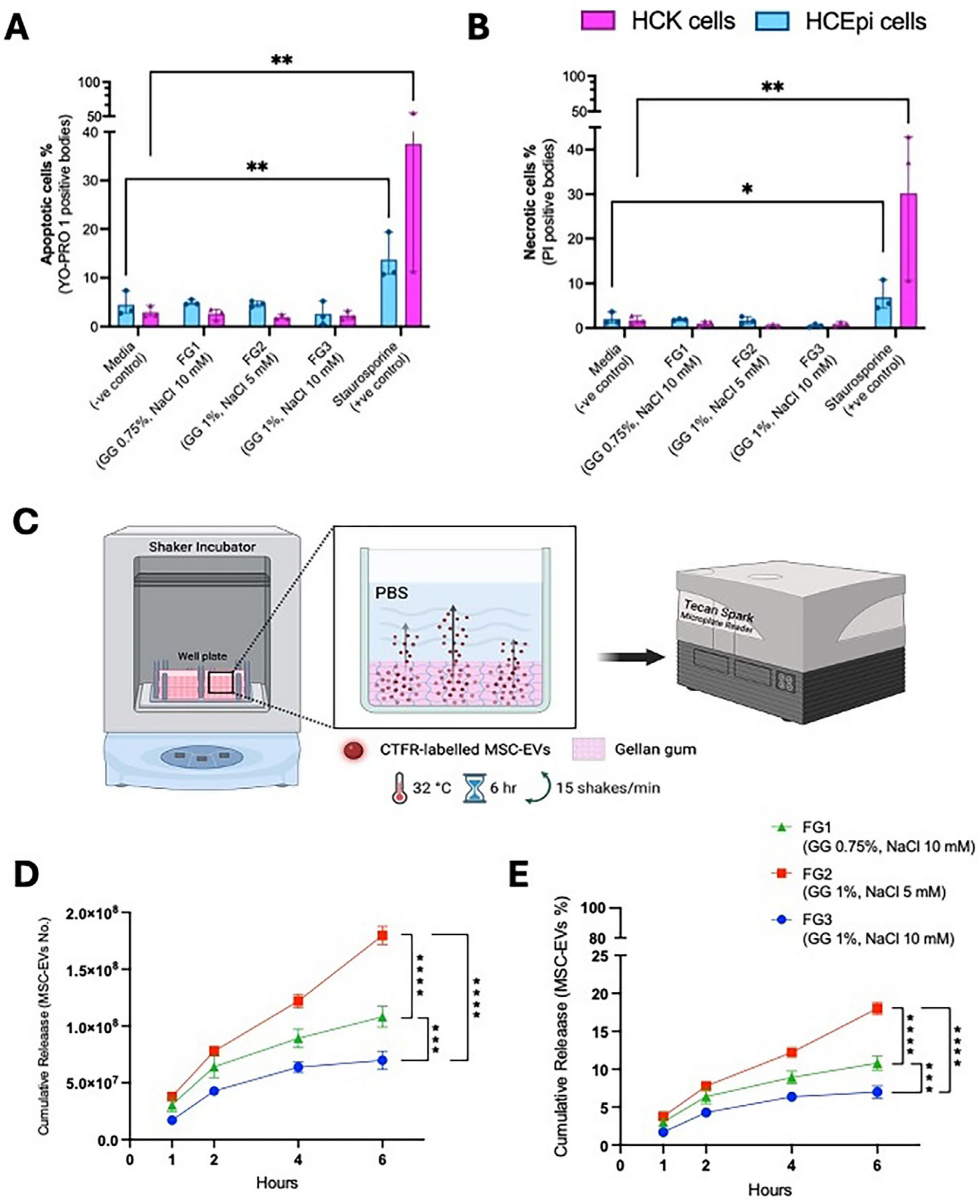
In vitro Biocompatibility and Release Kinetics of MSC‐EVs from selected Gellan Gum Fluid Gel Formulations. (A) Apoptosis induction by selected gellan gum fluid gel formulations (FG1, FG2, and FG3) in human corneal epithelial (HCEpi) and human corneal keratocyte (HCK) cells, assessed by YO‐PRO1 positive staining. (B) Necrotic effects of FG1, FG2, and FG3 on HCEpi and HCK cells, determined by PI positive staining. All data presented as mean ± 95% confidence interval (n = 3). Statistical analysis was performed using one‐way ANOVA with Dunnett's multiple comparisons test, where significance is denoted as *p = 0.0159, **p = 0.0067. (C) Schematic representation of the release assay setup. (D) Cumulative number of CTFR‐labelled cytokine‐licensed MSC‐EVs released from FG1, FG2, and FG3 at 1, 2, 4, and 6 hr timepoints. (E) Cumulative percentage release of MSC‐EVs from FG1, FG2, and FG3 at the same timepoints. All data presented as mean ± 95% confidence interval (n = 4). Statistical analysis was performed using one‐way ANOVA with Tukey's multiple comparisons test, where significance is denoted as ***p = 0.0003, ****p < 0.0001.

The selected gellan gum fluid gel formulations were evaluated for their in vitro biocompatibility by incubating HCK and HCEpi cells with FG1, FG2, and FG3. Cell viability was assessed by measuring necrosis through PI and apoptosis through YO‐Pro‐1 staining (Figure 3A,B). The total number of cells was determined using Hoechst staining. The results indicated no significant toxicity associated with FG1, FG2, or FG3 on HCEpi and HCK cells in terms of apoptosis (Figure 3A) and necrosis (Figure 3B) when compared to the negative control group, which received conditioned media. In contrast, significant effects were observed with Staurosporine treatment as the positive control, leading to a 10% (*p* < 0.05) and a 40% (*p* < 0.005) increase in apoptosis levels (Figure 3A), as well as a 5% (*p* < 0.05) and a 30% (*p* < 0.005) increase in necrosis levels in HCEpi and HCK cells, respectively (Figure 3B).

### 3.4 MSC‐EVs Cumulative Release From FG Systems

The in vitro release profile of CTFR‐labelled MSC‐EVs from selected gellan gum fluid gel formulations was evaluated over a 6‐h period at 32°C in 0.1 µm filtered PBS, using the Spark Cyto cell imager (Figure 3C), in order to identify the lead formulation for subsequent experiments. A fluorescence standard calibration curve was generated using a range of CTFR‐labelled MSC‐EVs (Figure S4A) resulting in the following concentration, Equation 2;

(2) Y=4.020e−005×X+234

Here, *Y* represents the fluorescence intensity measured by the instrument, and *X* denotes the concentration of CTFR‐labelled MSC‐EVs per mL of release media (Figure S4B).

The release profile of CTFR‐labelled MSC‐EVs from the fluid gel formulations FG1, FG2, and FG3 after 1 h was measured as 3.05 × 10^7^, 3.79 × 10^7^, and 1.7125 × 10^7^ MSC‐EVs, respectively (Figure 3D). These values correspond to 3.05%, 3.79%, and 1.7125% of the total MSC‐EVs loaded in each formulation (Figure 3E). Over time, the cumulative release of MSC‐EVs from FG1 (gellan gum 0.75%, NaCl 10 mM) increased to 6.3975% at 2 h, 8.9175% at 4 h, and 10.815% at 6 h. FG2 (gellan gum 1%, NaCl 5 mM) demonstrated a higher release rate, with cumulative values of 7.7825% at 2 h, 12.2075% at 4 h, and 17.9975% at 6 h. In contrast, FG3 (gellan gum 1%, NaCl 10 mM) exhibited a slower release, with cumulative values of 4.285% at 2 h, 6.3775% at 4 h, and 6.9875% at 6 h (Figure 3E). After 6 h, the total number of CTFR‐labelled MSC‐EVs released from the formulations showed statistically significant differences. FG2 released the highest number, with 1.7975 × 10^8^ MSC‐EVs (*p* < 0.0001 compared to FG1 and FG3). FG1 released 1.0805 × 10^8^ MSC‐EVs (*p* < 0.0001 compared to FG2, and *p* = 0.003 compared to FG3), while FG3 released the lowest number, 6.9875 × 10^7^ MSC‐EVs (*p* < 0.0001 compared to FG2, and *p* = 0.003 compared to FG1) (Figure 3D).

### 3.5 Released MSC‐EVs Uptake by HCEpi and HCK Cells

**FIGURE 4:**
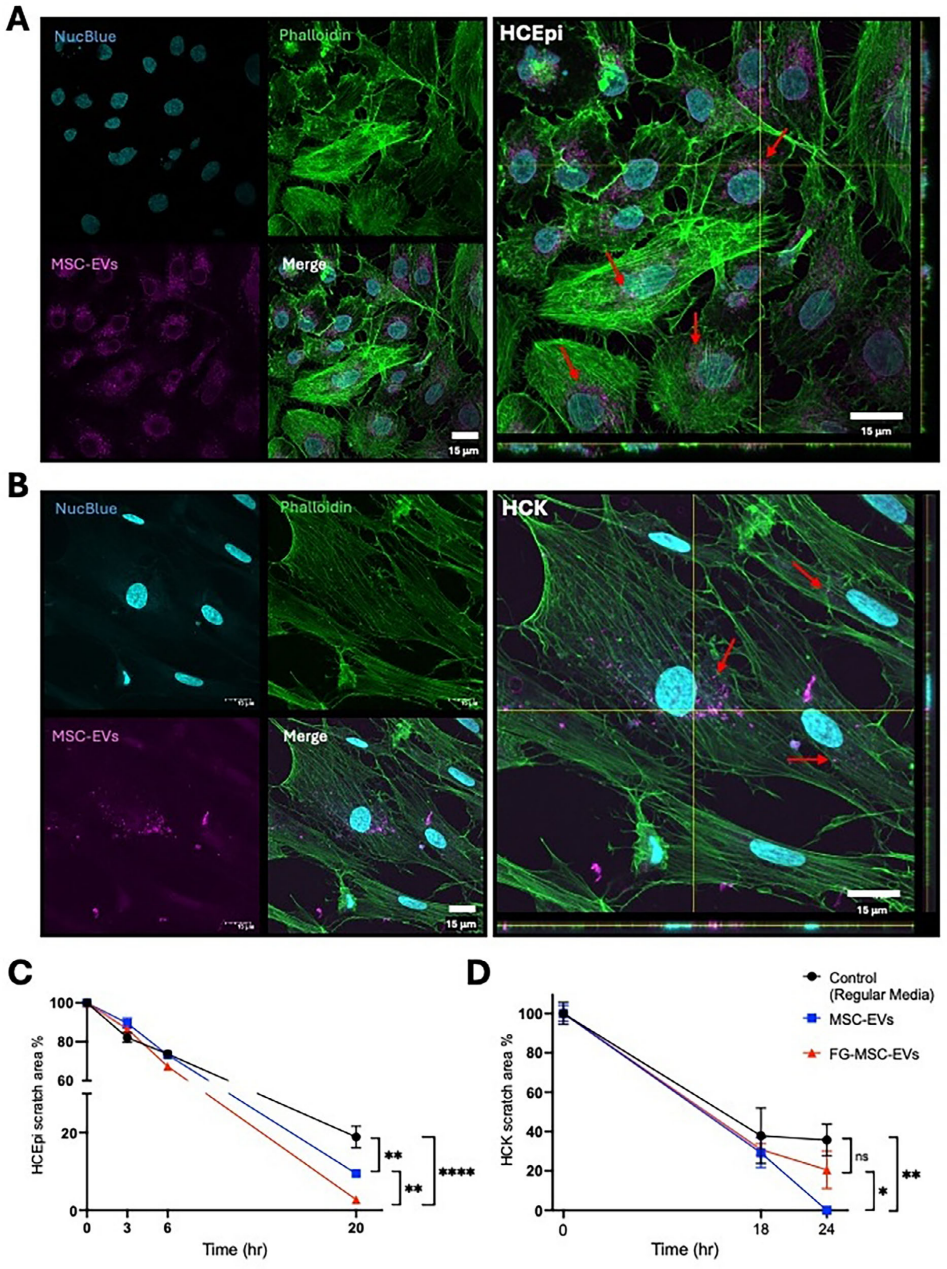
Uptake of fluorescently labelled cytokine‐licensed MSC‐EVs from gellan gum and their effect on wound closure in vitro in HCEpi and HCK cells. (A) Uptake of FG‐MSC‐EVs (magenta) by human corneal epithelial (HCEpi) cells, and (B) human corneal keratocyte (HCK) cells, with cytoskeleton stained by phalloidin (green) and nuclei counterstained with NucBlue (cyan). (C) Quantification of scratch area (%) in HCEpi cells and (D) HCK cells at different time points post‐scratch, following treatment with control medium, MSC‐EVs, or FG‐MSC‐EVs. Data are shown as mean ± 95% confidence interval (n = 3). Statistical analysis was performed using one‐way ANOVA with Tukey's multiple comparisons test, with significance indicated as *p < 0.0296 and **p = 0.0022 for HCK scratch closure, and ****p < 0.0001, **p = 0.0017, and **p = 0.0096 for HCEpi scratch closure.

To assess the functional impact of MSC‐EVs released from the gellan gum fluid gel on HCEpi and HCK cells, cells were treated with the lead formulation (FG2) loaded with fluorescently labelled MSC‐EVs (Figure S5), following by phalloidin and NucBlue staining and confocal microscopy imaging (Figure 4). The confocal microscopy images demonstrated that the MSC‐EVs were released from the FG2 and then were taken up by both HCEpi (Figure 4A) and HCK (Figure 4B) cells. The released MSC‐EVs were localized around the nucleolus in both cell types marked by the red arrows.

### 3.6 FG‐MSC‐EVs Improved the In Vitro HCK and HCEpi Cells Scratch Wound Healing Models

In vitro wound healing assays were conducted on HCK and HCEpi to evaluate the effects of MSC‐EVs released from FG on wound closure and cell migration (Figure 4C,D). No significant differences were observed between the MSC‐EVs treatment group and the control group (treated with standard culture medium), nor when compared to the FG‐MSC‐EVs group at 3 and 6 h post‐scratch in HCEpi cells and at 18 h post‐scratch in HCK cells. However, at 20 h post‐scratch in the HCEpi model, the FG‐MSC‐EVs treated group exhibited significantly greater wound healing, with only 2.7% of the scratch area remaining (*p* < 0.0001 vs. control; *p* = 0.0096 vs. MSC‐EVs group). In comparison, the residual scratch areas for the control and MSC‐EVs treated groups were 18.8% and 9.4%, respectively. The FG‐MSC‐EVs group also showed a significantly accelerated healing rate compared to the control (*p* = 0.0017, Figure 4C). In the HCK scratch assay, significant differences emerged at 24 h post‐scratch (Figure 4D). Wounds treated with MSC‐EVs were nearly fully healed (*p* = 0.0022 vs. control; *p* = 0.0296 vs. FG‐MSC‐EVs group). The remaining scratch area in the control group was 35.6%, while the FG‐MSC‐EVs group had 20.5% of the initial wound area remaining a difference that did not reach statistical significance (*p* = 0.0925, Figure 4D).

### 3.7 MSC‐EVs Accelerates Corneal Epithelial Wound Healing and Reduces Inflammation in Mice With Traumatic LSCD

**FIGURE 5:**
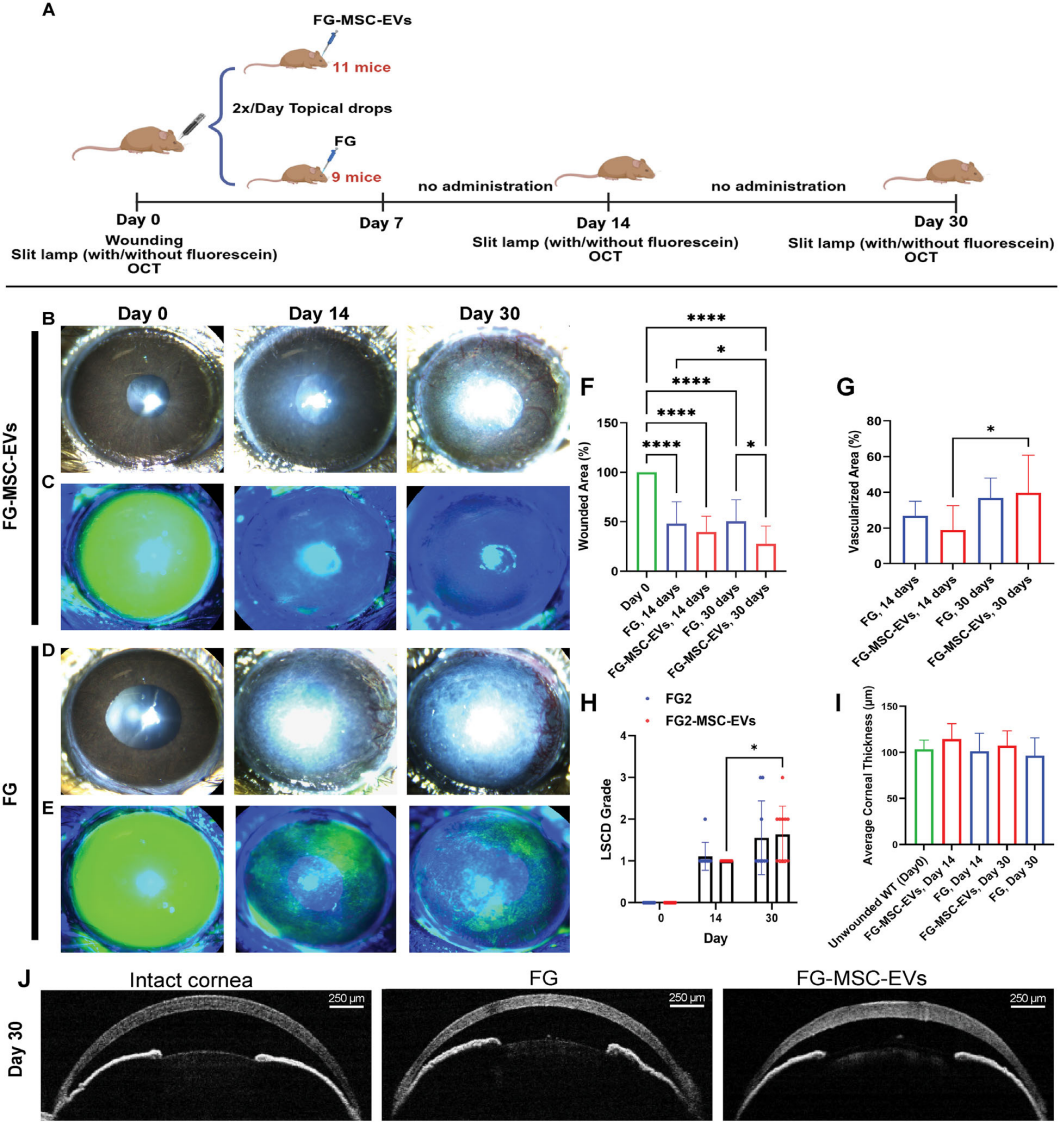
FG‐MSC‐EVs treatment promotes corneal wound healing in mice with traumatic LSCD. (A) schematic representation for the topical administration (B‐E) Representative images and quantitative analysis of corneal wound healing in WT mice treated with FG‐MSC‐EVs or FG alone. (B) Slit‐lamp images of WT corneas were captured at baseline (Day 0), after 14 days (7 days of FG‐MSC‐EVs treatment followed by 7 days without treatment) post‐wounding, and after a two‐week washout period (Day 30). (C) Fluorescein slit lamp images of WT corneas in the FG‐MSC‐EVs group after epithelial injury at Days 0, 14, and 30. (D) Slit‐lamp images of WT corneas were captured at baseline (Day 0), after 14 days (7 days of FG administration followed by 7 days without treatment) post‐wounding, and after a two‐week washout period (Day 30). (E) Fluorescein slit lamp images of WT corneas in the FG group at Days 0, 14, and 30 post‐injury. (F) Quantification of corneal wounded area expressed as the percentage of the wounded area relative to the total corneal surface area. (G) Quantification of corneal vascularization expressed as the percentage of the vascularized area relative to the total corneal surface area at different time points. (H) Comparison of LSCD grades between treatment groups at different time points. (I) Average corneal thickness measurements in FG and FG‐MSC‐EVs groups at different time points. (J) Representative OCT images from FG and FG‐MSC‐EVs groups.

Next, we evaluated the therapeutic efficacy of MSC‐EVs released from FG in a murine traumatic LSCD model. The therapeutic efficacy of FG and FG‐MSC‐EVs on corneal epithelial wound healing was quantitatively assessed using longitudinal slit‐lamp microscopy of the cornea (Figure 5B–E). Clinical observations revealed reduced corneal scarring and fluorescein staining in the FG‐MSC‐EVs group compared with the FG group (Figure 5B–E). Notably, a few animals (three from the FG group and one from the FG‐MSC‐EVs group) reached humane endpoints during the course of eye drop administration due to the aggressive full‐corneal wound induced and were excluded from subsequent analyses. Statistical analysis of fluorescein slit‐lamp images revealed a significant reduction in corneal wound area in the FG‐MSC‐EVs group compared with the FG‐only group (*p* = 0.02, Figure 5F). At the 14‐day time point, the FG‐MSC‐EVs group exhibited a trend toward reduced corneal vascularization area and lower standard vessel grading compared with the FG group; however, this difference did not reach statistical significance. Conversely, at the 30‐day time point, a significant increase in both vascularized area (*p* = 0.01, Figure 5G) and LSCD grading (*p* = 0.03, Figure 5H) was observed in the FG‐MSC‐EVs group. Representative OCT images and average corneal thickness measurements showed that the biomaterial had no adverse effect on corneal thickness and did not cause any thinning (Figure 5I,J).

**FIGURE 6:**
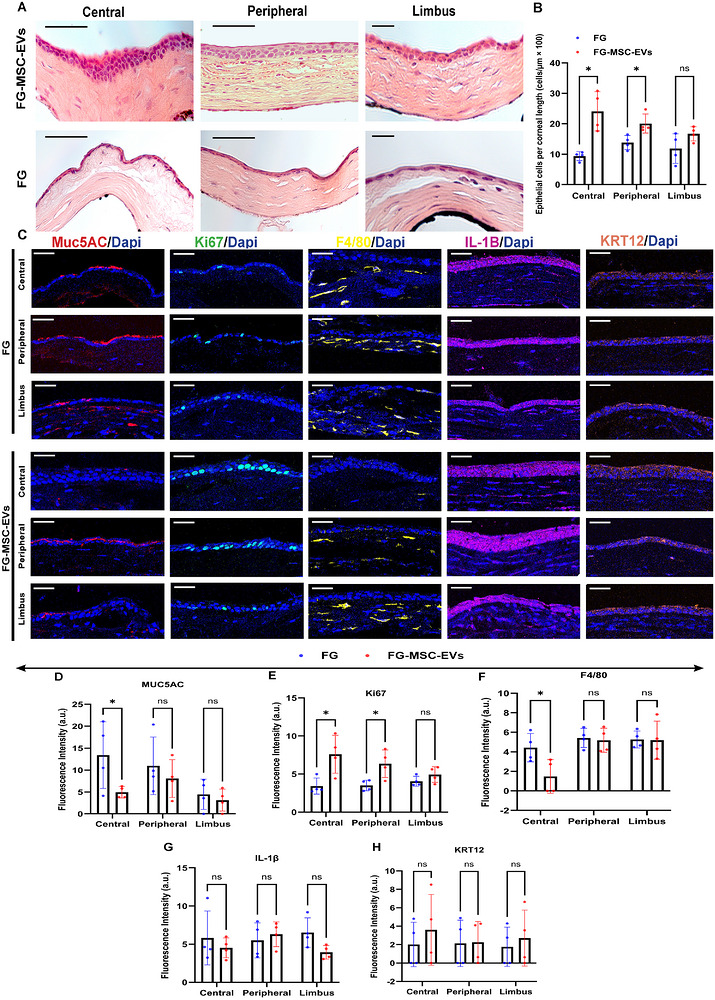
FG‐MSC‐EVs treatment demonstrates higher epithelial cell density and potentially reduce inflammation in mice with traumatic LSCD. (A) Representative H&E‐stained images of the central cornea, peripheral cornea, and limbal regions from the FG and FG‐MSC‐EVs groups. (B) Quantification of epithelial cell number per corneal length in the central and peripheral regions.(C) Representative immunofluorescence images showing MUC5AC, Ki‐67, F4/80, IL‐1β, and KRT12 expression in the central, peripheral, and limbal regions from both groups.(D–H) Quantification of fluorescence intensity for MUC5AC (D), Ki‐67 (E), F4/80 (F), IL‐1β (G), and KRT12 (H) in the corresponding regions of the cornea. Scale bars = 50 µm.

A total of four corneas from four different mice in the FG group and four corneas from four different mice in the FG‐MSC‐EVs group were analysed by H&E staining and immunofluorescence at day 30 post‐injury. H&E staining revealed a significantly greater epithelial cell density in both the central (*p* = 0.02) and peripheral (*p* = 0.02) regions of corneas from the FG‐MSC‐EVs group compared with the FG group (Figure 6A,B). In addition, the central corneal stroma of FG‐MSC‐EVs‐treated mice exhibited a more organized architecture and better‐preserved tissue integrity relative to FG controls (Figure 6A). Quantitative fluorescence intensity analysis demonstrated that conjunctival MUC5AC expression was significantly higher in the central cornea of FG mice compared with FG‐MSC‐EVs mice (*p* = 0.04), with no significant differences observed in the peripheral or limbal regions (Figure 6C,D). Proliferative activity, indicated by Ki‐67 staining, was sparse in the FG group but significantly elevated in the central (*p* = 0.04) and peripheral (*p* = 0.04) corneal epithelium in the FG‐MSC‐EVs‐treated group (Figure 6C,E). The macrophage marker F4/80 showed reduced expression in the central cornea of the FG‐MSC‐EVs‐treated group at day 30 post‐injury compared with FG controls (*p* = 0.04), whereas no differences were detected in the peripheral or limbal regions (Figure 6C,F). The inflammatory marker IL‐1β was abundantly expressed in the central cornea and stroma of both groups, with no significant intergroup differences (Figure 6C,G). Notably, KRT12 was expressed in an epithelial layer across all corneal regions, with no significant differences between groups; however, a trend toward higher expression was observed in the FG‐MSC‐EVs group compared to the FG group in the central cornea. (Figure 6C,H).

## 4 Discussion

Mesenchymal stromal cell‐derived extracellular vesicles (MSC‐EVs) are increasingly recognized for their regenerative and immunomodulatory functions, with clear advantages over conventional cell‐based therapies [17, 57]. In the field of ophthalmology, for example, MSC‐EVs have demonstrated a remarkable ability to speed up wound healing in in vitro, *ex vivo*, cornea‐on‐a‐chip, and pre‐clinical models [26, 58, 59, 60, 61]. Importantly, the therapeutic potential of MSC‐EVs can be further amplified through pre‐activation or licensing [19]. Licensing with pro‐ and anti‐inflammatory cytokines shown to be a direct strategy to activate the immunomodulatory machinery of MSCs by mimicking the inflammatory microenvironment encountered in vivo [19] and upregulating key immunoregulatory mediators [23] in contrast to physical stimulation strategies such as 3D culture systems [62] which mainly serves as a supportive approach to enhance general MSC functionality.

In previous works [24], we have shown that licensing MSCs with a combination of IFN‐γ and TGF‐β1 boosts the immunoregulatory power of their secreted EVs. In addition, cytokine‐licensed MSC‐EVs were found to enhance corneal epithelial wound healing more effectively than naïve MSC‐EVs [27]. However, their therapeutic application remains constrained by delivery challenges, particularly when administered topically, as tear film turnover and blinking rapidly wash EVs from the corneal surface [63]. Addressing this limitation requires delivery systems that can prolong EV residence and release at the target site. Various strategies have been explored to enhance the retention of therapeutics on the corneal surface, such as contact lenses [64], injections [65], or dressing sheets [66]. While these approaches can prolong contact time, they are invasive and often disliked by patients, or in severe injuries can increase the risk of complications, such as microbial keratitis [66, 67, 68].

In this study, gellan gum fluid gels were investigated as a delivery platform for MSC‐EVs. Fluid gels are characterized by shear‐thinning properties, allowing them to behave as weak gels at rest but transition to a more fluid state during application [37]. The fluid gel rapidly restructures after topical application, forming a soft elastic network on the ocular surface immediately while remaining optically clear. It provides uniform corneal coverage with high retention, remaining detectable on rat eyes for up to 120 min without localized pooling. Acting as a temporary occlusive bandage, the gel can stay on the ocular surface for around 2 h and is gradually cleared naturally by blinking over 4–6 h [37, 38, 69]. This rheological responsiveness provides advantages for ocular use, the formulation spreads easily across the corneal surface during blinking, then re‐establishes a gel‐like structure that prolongs residence time and reduces clearance. Such behavior supports the rationale for testing this system with MSC‐EVs.

All gellan gum fluid gel formulations exhibited desirable shear‐thinning behavior, enabling flow under shear while retaining structural integrity at rest (Figure S3B). This property is critical for ocular applications, allowing easy spreading during blinking and maintaining viscosity between blinks to resist precorneal drainage which prolong residence time on the ocular surface [37, 70, 71, 72, 73]. The increased retention time also relies on mucoadhesive properties of the gellan gum fluid gel without permanent covalent attachment, supporting tissue remodelling while remaining removable through natural blinking in contrast with tissue adhesive systems such as oxidized polysaccharide/chitosan formulations [74] that provide strong tissue adhesion via covalent bonding. Although tissue adhesion through covalent binding can be advantageous in some contexts, they may risk persistent adherence and formation of a physical barrier that could hinder cell migration, tissue repair, and inconvenient patient experience depending on application. Increasing gellan gum concentration raised both zero‐shear viscosity (η_0_) and critical shear stress (1/K), indicating denser polymer networks and stronger intermolecular associations aligns with previous findings showing enhanced chain entanglement and junction‐zone density [75, 76]. Crosslinkers further increased viscosity through ionic interactions between polymer chains, stabilizing the network at low shear rates, improving ocular retention [71, 77, 78].

Small‐deformation rheological analysis showed that polymer concentration had a greater effect than crosslinker content on the storage modulus (G′), confirming that gel elasticity is primarily governed by network density [75, 76]. Similar concentration‐dependent increases in G′ have been reported for gellan and other polysaccharide hydrogels [77, 79]. Clinically, higher G′ values improve structural stability on the ocular surface but must be balanced to maintain comfort and spreadability. Amplitude and frequency sweeps identified crossover points between G′ and G″, representing the transition from elastic to viscous behavior. These transitions mirror the yielding events occurring during blinking and have been linked to controlled drug release as shear disrupts the network to facilitate diffusion [37, 70, 76].

To select and narrow down the fluid gel formulations for ocular delivery of the MSC‐EVs, the gellan gum fluid gel formulations were compared to the range Grover and colleagues obtained from commercially available eyedrops and ointments and ophthalmologists opinions [55]. Among the tested formulations, FG1 (0.75% gellan gum, 10 mM NaCl) achieved viscosity within the 3–5 Pa·s, the selection range for topical ophthalmic delivery, consistent with prior recommendations and the commercial products properties [55, 72]. Within the linear viscoelastic region, FG2 (1% gellan gum, 5 mM NaCl) and FG3 (1% gellan gum, 10 mM NaCl) achieved the most favourable balance between elasticity and shear‐thinning flow (Figure 2B). This rheological balance is ideal for ocular delivery, providing cohesive strength for retention and controlled deformation under shear to permit EV release and tear clearance. Comparable observations have been reported in gellan‐based hydrogels for sustained ocular delivery [37, 38, 69, 71]. Overall, tuning polymer concentration and ionic strength offers a robust strategy for achieving formulations with desirable viscoelastic behavior for MSC‐EVs ocular delivery. These FG eye drop formulations dynamically adapt to blinking forces while recovering their structure and viscosity between cycles, promoting ocular surface retention and patient comfort.

Safety and tolerability are essential considerations for translation of topical formulations. Our data demonstrated that FG1, FG2, and FG3 did not induce apoptosis or necrosis in corneal epithelial or keratocyte cells (Figure 3). This absence of cytotoxicity supports their suitability for further preclinical testing and is consistent with previous reports on the biocompatibility of gellan gum‐based gels in ocular systems [37, 38]. Controlled release of MSC‐EVs was observed from the fluid gel matrix using fluorescence‐based methods to minimize the biomaterials micro/nanoparticles cross‐interaction [80] compared to other methods such as protein‐based assays [62, 74], NTA, or immunobeads assay [79]. The polymer and crosslinker concentrations strongly influence release kinetics, as the lower concentration of cross linker resulted in weaker interaction between the sheared microgels and resulting in easier migration and release of the MSC‐EVs. FG2 demonstrated the most favourable release characteristics, providing sustained EV delivery with approximately 18% release at 6 h, significantly higher than FG1 and FG3 (Figure 3). Therefor FG2 was selected as the leading gellan gum fluid gel formulation for the subsequent in vitro and in vivo studies. The lack of a burst release suggests uniform EV incorporation within the gel microstructure, avoiding rapid loss of surface‐entrapped EVs during early time points [81]. This controlled release profile contrasts with the rapid clearance reported in studies applying free MSC‐EVs topically, which necessitated repeated dosing to maintain therapeutic activity [26, 82, 83]. Sustained release represents an important step toward reducing dosing frequency and improving therapeutic efficacy. Fluorescent imaging confirmed that EVs were homogeneously distributed throughout the fluid gel system shown in Figure S5, supporting the observed release characteristics. Uptake assays demonstrated effective internalization of EVs by both epithelial and stromal cells (Figure 4A,B), consistent with earlier findings showing that hrDecorin, a wound‐healing molecule [37], and EVs [79] preserve activity when delivered via gellan gum matrices. Functional in vitro assays further confirmed preservation of EV activity. In scratch wound models, epithelial cells treated with FG2‐delivered EVs exhibited accelerated repair compared to untreated controls, supporting the hypothesis that the fluid gel microenvironmental support may enhance EV‐mediated healing (Figure 4C). This aligns with prior observations that EVs maintain wound‐healing capabilities after encapsulation [79]. Interestingly, in keratocyte assays, EVs delivered via the fluid gel system produced slower wound closure compared with EVs applied directly (Figure 4D). Uptake analysis indicated reduced internalization of gel‐released EVs by keratocytes relative to epithelial cells (Figure 4A,B), suggesting that cell‐type specific interactions influence therapeutic outcomes.

The gellan gum fluid gel's shear‐thinning behavior reduces application effort, enhances surface retention, and improves patient comfort and tolerability. Unlike viscous or implant‐based systems, it leaves no residual material upon discontinuation, and its favourable physicochemical and safety profile, including low irritation, good stability, and prolonged ocular residence time [84], may facilitate regulatory acceptance as an incremental improvement to existing topical therapies. In contrast, photocured or implantable hydrogels [62] require in situ curing or surgical placement, bright‐light activation, and are not readily reversible. As they are primarily designed for structural support, they are less suited to non‐invasive, patient‐friendly topical therapy [85]. Both fluid gel systems and other advanced ocular delivery platforms, however, remain largely at the preclinical or early translational stage, and conclusions regarding patient comfort, usability, and long‐term clinical adoption await systematic clinical evaluation.

In vivo, MSC‐EVs exerted a beneficial effect on corneal epithelial healing in a model of total LSCD by promoting epithelial regeneration and maintaining epithelial identity. Among the markers examined, KRT12 is a specific differentiation marker for mature corneal epithelial cells [86], MUC5AC is a well‐established marker of conjunctivalization, and Ki67 is a proliferation marker for corneal re‐epithelialization [52, 86, 87]. Reduced MUC5AC expression and retained KRT12 expression in the central cornea of the FG‐MSC‐EVs group suggests that MSC‐EVs helped prevent conjunctival overgrowth, in contrast to the FG group, where corneal epithelium adopted a conjunctival phenotype. Enhanced cellular proliferation and epithelial regeneration were evident in the FG‐MSC‐EVs group, indicated by increased Ki67 expression and greater epithelial cell density in a normal, multilayered structure. Together, these findings imply that MSC‐EVs stimulated proliferative activity within the basal epithelial layer, contributing to faster and more organized epithelial recovery following injury. The widespread expression of IL‐1β observed across the cornea in both the FG and FG‐MSC‐EV groups suggests active participation of this cytokine in the wound healing response following injury. IL‐1β is a key mediator that regulates multiple aspects of corneal repair and angiogenesis [88]. This is consistent with the observed increase in LSCD grading and vascularized area in both groups, that was however unabated with treatment, reflecting the total removal of limbal stem cells in this model. Elevated IL‐1β and IL‐6 levels correlate with increased F4/80^+^ macrophage infiltration in the stroma [86], consistent with macrophages being a major source of these cytokines [89]. Notably, macrophage infiltration was markedly reduced in central cornea of the FG‐MSC‐EVs group, suggesting that MSC‐EVs suppress macrophage recruitment, which together with proliferation of an epithelial phenotype, indicates an improved healing response in LSCD, but not one that can fully replace limbal stem cells in cases of total deficiency, at least with the treatment regimen given in this study. This study was limited to two animal groups receiving either FG or FG‐MSC‐EVs, considering the goals of this study, well‐established therapeutic effects of MSC‐EVs on corneal injuries [90, 91, 92], animal welfare principles, ethical considerations, and to reduce the unnecessary suffering given the known effect of the model alone on corneal phenotype, as documented in prior studies [93]. Further investigation, however, including different disease models and additional treatment groups such as MSC‐EV only and using appropriate negative control groups are needed to confirm the advantages of a delivery system providing EVs directly to the injury site and enhanced therapeutic effects we observed, in a broader context.

## 5 Conclusion

In conclusion, this study highlights the therapeutic potential of cytokine‐licensed MSC‐EVs when delivered via a gellan gum fluid gel system to the ocular surface. The selected gel formulation enabled sustained ocular surface residence and controlled MSC‐EVs release, resulting in enhanced cellular uptake and preservation of MSC‐EV bioactivity. Importantly, MSC‐EVs released from the formulation promoted significant tissue repair and accelerated wound healing in both in vitro and pre‐clinical models, demonstrating their regenerative efficacy. The gellan gum matrix served as a biocompatible carrier that supported MSC‐EVs stability and delivery, amplifying their biological function. Overall, these findings underscore the promise of MSC‐EV therapy for ocular diseases and position gellan gum fluid gels as a practical delivery vehicle to translate this potential into further pre‐clinical and clinical applications.

## Conflicts of Interest

The authors declare no conflict of interest.

## Supporting information




**Supporting File**: adhm71402‐sup‐0001‐SuppMat.docx.

## Data Availability

The data that support the findings of this study are available from the corresponding author upon reasonable request.
