# Supplementary material for: Gellan Gum Fluid Gel System for Controlled‐Delivery of Cytokine‐Licensed MSC‐EVs to Enhance Corneal Repair in Limbal Stem Cell Deficiency
Source: Adv Healthc Mater. 2026 Jun 30;15(29):e71402. doi: 10.1002/adhm.71402 (PMC13447925; doi:10.1002/adhm.71402)
Supplement: Supplementary file 1 [file ADHM-15-0-s001.docx]

## **Supplementary Materials**

**Method S1. MSCs culture, cytokine licensing, and preparation for MSC-EVs isolation.**

Human bone marrow mesenchymal stromal cells (MSCs) were isolated from the bone marrow of three healthy volunteers at Galway University Hospital, following ethical approval and written informed consent. MSCs were cultured according to a standardized protocol at a density of 5,000 cells/cm² in T175 flasks, using MSC growth medium composed of MEM α with GlutaMAX™ Supplement (Gibco, ThermoFisher Scientific, cat#: 32561029), 10% FBS (GE Healthcare, FisherScience, Ref: 10309433), 1% penicillin-streptomycin (Gibco, ThermoFisher Scientific, cat#: 15140122), and 1 ng/mL FGF-β (Gibco, ThermoFisher Scientific, cat#: 100-18B-10UG). Cells were incubated at 37°C and 5% CO2, with media changes every three days. Upon reaching 90% confluency, the culture medium was removed, and cells were washed twice with PBS (Gibco, ThermoFisher Scientific, cat#: 14190094). They were then incubated in xeno-free cytokine licensing growth medium supplemented with 50 ng/mL TGF-β1 (Bio-Techne/R&D Systems, Cat#: 7666-MB-005) and 50 ng/mL IFN-γ (PeproTech, ThermoFisher Scientific, Cat#: 315-05) for 72 hours at 37°C and 5% CO2. After incubation, the conditioned medium was collected and processed to remove cell debris and apoptotic bodies through centrifugation at 400 x g for 10 minutes followed by 2,000 x g for 30 minutes. The resulting medium was concentrated using Amicon Ultra-15 centrifugal filter units with 100 kDa MWCO filters at 3,000 x g for 30 minutes.

**Figure S1**	Fluorescein and slit-lamp images of a mouse cornea before and at 14 and 30 days post-epithelial removal without treatment, indicating a severe LSCD phenotype induced by total epithelial removal.

**Figure S2**	Cytokine-licensed MSC-EVs characterization controls. (A) NTA analysis of the QC-100 nm beads, (B) flow cytometry control panel, Apogee calibration beads, filtered PBS, unstained MSC-EVs, and stained MSC-EVs.

**Figure S3**	Gellan gum fluid gel formulations characterization. (A) transparency analysis, (B) viscosity profile, (C) frequency sweep analysis, (D) amplitude sweep analysis – (i) 0.5% gellan gum fluid gels, (ii) 0.75% gellan gum fluid gels, (iii) 1% gellan gum fluid gels, (iv) 1.5% gellan gum fluid gels.

**Figure S4**	CTFR-labelled Cytokine-licensed MSC-EVs standard calibration. (A) table indicating the number of CTFR-labelled Cytokine-licensed MSC-EVs in each sample, (B) standard calibration curve and the standard equation of the fluorescence intensity of various concentrations of CTFR-labelled Cytokine-licensed MSC-EVs. All data presented is the average on n = 3, with error bars demonstrating the 95% confidence interval.

**Figure S5**	Fluorescently labelled cytokine-licensed MSC-EVs distribution inside the gellan gum fluid gel matrix. Gellan gum fluid gel with fluorescently labelled cytokine-licensed MSC-EVs (magenta), (A) 2D view, (B) Z-stack view, and (C) 3D view.
